# Trophic niches of Collembola communities change with elevation, but also with body size and life form

**DOI:** 10.1007/s00442-023-05506-7

**Published:** 2024-01-24

**Authors:** Johannes Lux, Zhijing Xie, Xin Sun, Donghui Wu, Stefan Scheu

**Affiliations:** 1 J.-F. Blumenbach Institute of Zoology and Anthropology, University of Göttingen, Untere Karspüle 2, Tierökologie, 37073 Göttingen, Lower Saxony Germany; 2 Key Laboratory of Wetland Ecology and Environment, Northeast Institute of Geography and Agroecology, Chinese Academy of Sciences, Changchun, China; 3 Key Laboratory of Vegetation Ecology, Ministry of Education, Northeast Normal University, Changchun, China; 4 Key Laboratory of Urban Environment and Health, Institute of Urban Environment, Chinese Academy of Sciences, Xiamen, China; 5 Jilin Provincial Key Laboratory of Animal Resource Conservation and Utilization, Northeast Normal University, Changchun, China; 6 Centre of Biodiversity and Sustainable Land Use, University of Göttingen, Göttingen, Germany

**Keywords:** Decomposers, Forest ecology, Functional ecology, Soil food web, Stable isotopes

## Abstract

Climate change will likely increase habitat loss of endemic tree species and drives forest conversion in mountainous forests. Elevation gradients provide the opportunity to predict possible consequences of such changes. While species compositions of various taxa have been investigated along elevation gradients, data on trophic changes in soil-dwelling organisms are scarce. Here, we investigated trophic changes of the Collembola communities along the northern slope of Changbai Mountain, China. We sampled Collembola in primary forests at seven elevations (800–1700 m asl). We measured individual body lengths and bulk stable isotopes on species level. We further categorized Collembola species into life forms. The community-weighted means of Δ^15^N and Δ^13^C values as well as minimum Δ^15^N values and isotopic uniqueness of Collembola communities increased with increasing elevation, while the range of Δ^15^N values decreased. Maximum and minimum of Δ^13^C values differed between elevations but showed no linear trend. Further, Δ^15^N values of Collembola species occurring across all elevations increased with elevation. Changes in Δ^15^N values with elevation were most pronounced in hemiedaphic species, while Δ^13^C values increased strongest with elevation in euedaphic species. Δ^15^N values increased with decreasing body size in hemiedaphic and euedaphic species. Overall, the results suggest that Collembola species functioning as primary decomposers at lower elevations shift towards functioning as secondary decomposers or even predators or scavengers at higher elevation forests. The results further indicate that access to alternative food resources depends on Collembola life form as well as body size and varies between ecosystems.

**Supplementary Information:**

The online version contains supplementary material available at 10.1007/s00442-023-05506-7.

## Introduction

Global climate change strongly affects forest systems across the world by accelerating extreme climate events resulting in increased tree mortality (Kharuk et al. 2017, 2021; Obladen et al. 2021). Among worldwide forest biomes mountain forest account for 23% of forest cover (Price et al. 2011). Climate change likely has detrimental consequences for mountain forests through climate driven forest conversion (Albrich et al. 2020), decreased tree growth (Matskovsky et al. 2021) and shrinking boundaries of endemic tree species (Dakhil et al. 2021). Further, Albrich et al. (2020) predicted climate change driven replacement of coniferous by deciduous forests in the European Alps. Changes in tree species composition can affect biodiversity in many taxa, the direction of the response, however, depends on taxon (Leidinger et al. 2021). Investigating elevation gradients in natural forests may therefore help to predict the response of different forest-dwelling taxa to changing climate and the resulting change in tree species composition. Changbai Mountain in North-Eastern China represents such a gradient as it comprises undisturbed montane forests including the transition between broadleaf-coniferous mixed and pure coniferous forests (Liu 1997; Tang et al. 2011).

Responses of a variety of plant, animal and microbial taxa across elevation gradients have been studied (Samson et al. 1997; Blake and Loiselle 2000; Hodkinson 2005; McCain 2005; Bhardwaj et al. 2011), including belowground animal communities (Fischer et al. 2014; Maraun et al. 2014; Bokhorst et al. 2018; Xie et al. 2022; Pan et al. 2023a). However, few studies focus on changes in trophic niches across elevation gradients, where data on belowground communities are especially scarce. Fischer et al. (2014) as well as Pan et al. (2023b) found varying responses in trophic niches of oribatid mites with elevation, indicating changes in soil food web interactions along elevation gradients.

Like oribatid mites, Collembola are among the most abundant soil microarthropods in temperate forests (Seastedt 1984), where they occupy a wide range of trophic levels (Scheu and Falca 2000; Chahartaghi et al. 2005). They predominantly feed on litter resources (Rusek 1998) and microorganisms, in particular fungi (Caravaca and Ruess 2014; Pollierer and Scheu 2021), thereby directly and indirectly affecting litter decomposition and nutrient cycling. The contribution of soil mesofauna to litter decomposition has been found to depend on litter characteristics, such as nutrient concentrations, which differ between tree species (Fujii et al. 2018). At Changbai Mountain, litter characteristics such as C/N ratio have been shown to vary strongly between the lower elevation broadleaf-coniferous mixed forests, where C/N ratio were rather low, and high elevation pure coniferous forests with higher C/N ratios. Additionally, microbial biomass increases with increasing elevation at Changbai Mountain (Lux et al. 2022). Therefore, direct contributions of the Collembola community to decomposition of litter might be higher at lower elevations, while Collembola may feed more intensively on the more abundant microbial resources at higher elevations. Collembola in forest ecosystems have been found to predominantly rely on saprotrophic fungi (Pollierer and Scheu 2021; Li et al. 2022); consequently, the trophic positions of Collembola communities may increase with elevation.

**Fig. 1:**
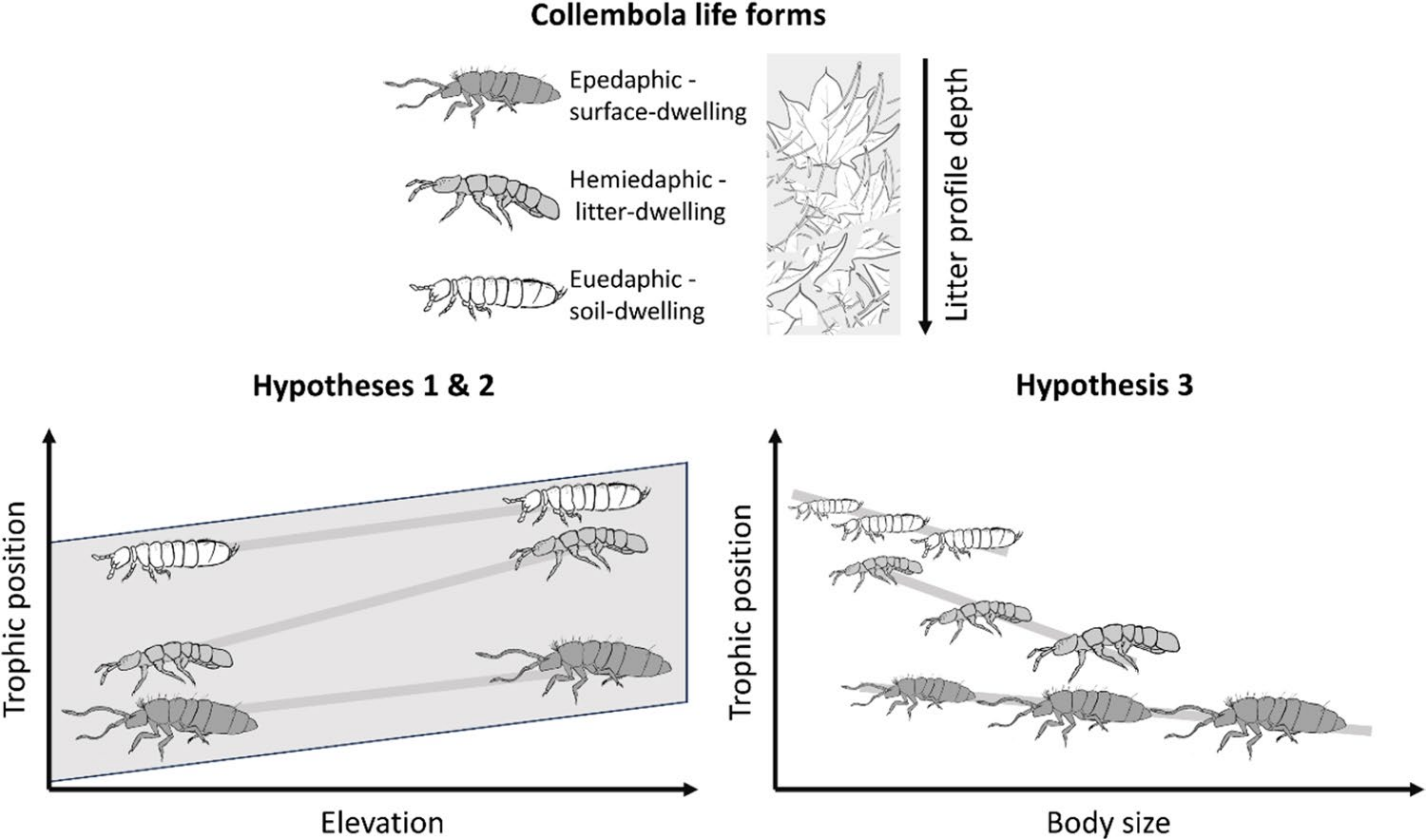
Conceptualized relationship between trophic positions of Collembola communities, life forms and elevation (Hypotheses 1 and 2), and between trophic positions of life forms and body size (Hypothesis 3)

Resources in forest soil systems are known to vary across microhabitats in the litter/soil matrix (Erktan et al. 2020). The ability of Collembola to access different resources within the litter/soil matrix across elevation gradients may mitigate but also aggravate trophic responses to elevation. Access to resources by Collembola may vary in particular with Collembola life forms (Gisin 1943; Rusek 2007). While epedaphic (surface-dwelling) Collembola predominantly colonize the litter surface, hemiedaphic (litter-dwelling) and euedaphic (soil-dwelling) Collembola can forage deeper in soil (Fujii and Takeda 2012). Potapov et al. (2016) found the trophic positions of life forms to increase from epedaphic to hemiedaphic to euedaphic Collembola, reflecting the different foraging strategies in the soil matrix (Fig. 1). Hemiedaphic Collembola, for instance, can forage on top of the litter as well as in deeper layers. This may provide hemiedaphic species access to a larger variety of resources compared to e.g., epedaphic species and therefore allows increased trophic plasticity along environmental gradients. Even though body size is among the traits differing between the three life forms in general (Rusek 2007), within life form body size variations may further influence foraging strategies. For example, the ability of euedaphic Collembola species to access smaller soil pores deeper in soil is likely determined by their body size (Rusek 2007). Stable isotopes are the major method for characterizing trophic niches in soil food webs (Potapov et al. 2019a; Maraun et al. 2023). Litter-normalized ^15^N/^14^N ratios (Δ^15^N) of consumers are commonly used to determine trophic positions, while litter-normalized ^13^C/^12^C ratios (Δ^13^C) provide information on the basal resource used by soil animals (Klarner et al. 2014; Potapov et al. 2019a; Maraun et al. 2023). However, especially when investigating organisms varying in vertical distribution within the litter/soil matrix, both Δ^15^N and Δ^13^C values can provide additional information about the resources used since organic matter in deeper soil layers is enriched in ^15^N and ^13^C compared to litter material on top of the soil (Garten et al. 2000; Ponsard and Arditi 2000; Wallander et al. 2004; Potapov et al. 2019a), which is likely due to the accumulation of microbial residues.

Bulk stable isotopes of nitrogen and carbon further allow calculation of isotope metrics to characterize trophic niches of consumer in more detail (Villéger et al. 2008; Cucherousset and Villéger 2015). In this study we use abundance weighted and unweighted bulk stable isotope metrics to characterize variations in trophic niches of Collembola communities (measured at species level) at Changbai Mountain (China). We further investigate changes in trophic niches of different Collembola life forms and species with elevation. Additionally, we study variations in trophic niches of Collembola with body size. We hypothesized that (1) abundance weighted Δ^15^N values linearly increase with elevation, while Δ^13^C values only slightly increase with elevation; (2) Δ^15^N values strongly and Δ^13^C values slightly differ among Collembola life forms, being highest in euedaphic and lowest in epedaphic Collembola, while the changes in Δ^15^N values with elevation are strongest in hemiedaphic Collembola; and (3) smaller Collembola occupy higher trophic positions as reflected in high Δ^15^N values and this relationship is most pronounced in hemiedaphic Collembola.

## Material and methods

### Study site and sampling

**Fig. 2:**
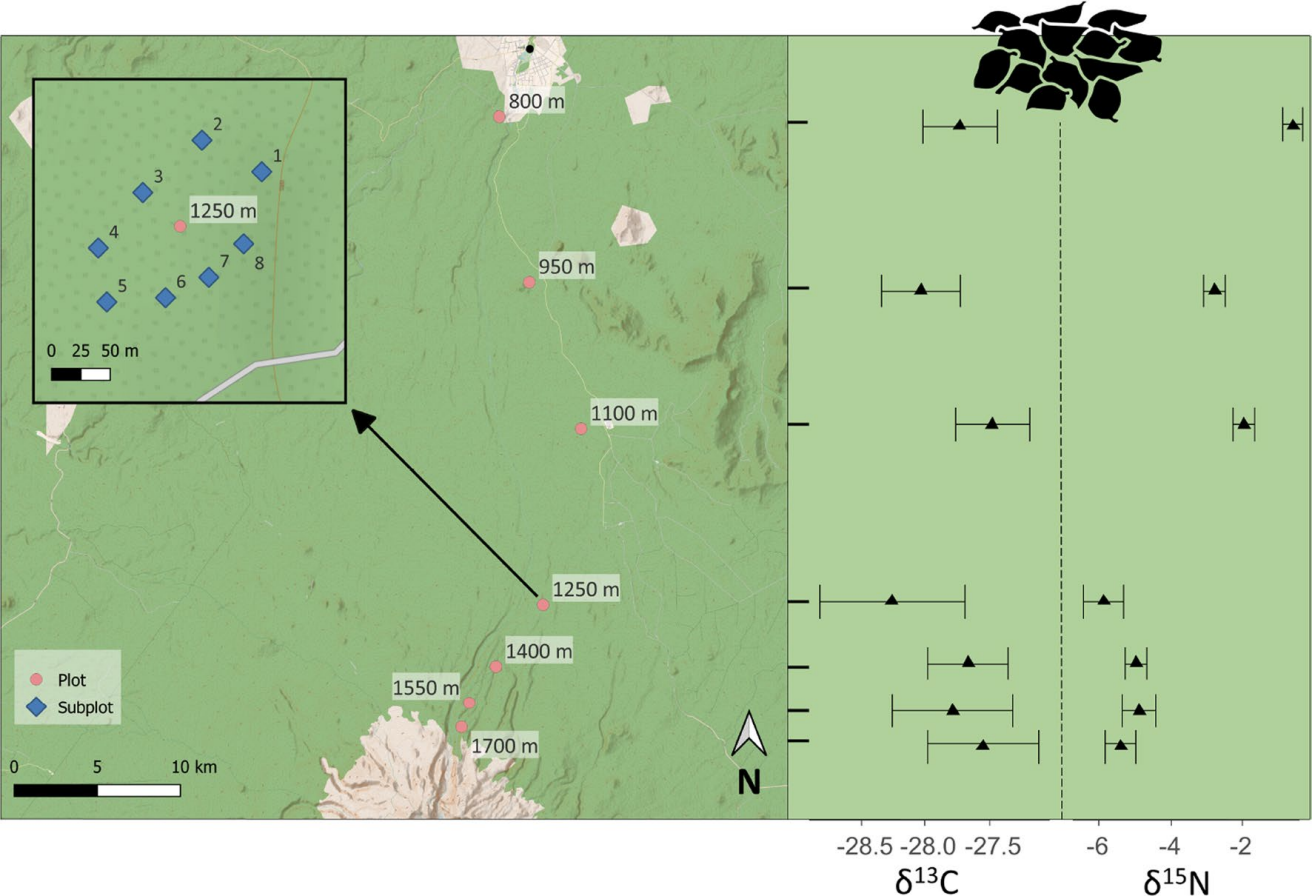
Map of the sampled transect at the northern slope of Changbai Mountain, China. The enlarged section displays the subplot structure for one of the elevations. The right panel displays the natural abundances of ^13^C and ^15^N of the litter for each elevation (means ± SD). Map data from “OpenTopoMap” (Erhardt et al. 2022)

The study was conducted at the northern slope of Changbai Mountain at the borders of the Chinese provinces Jilin and Liaoning to North Korea within the boundaries of the Changbaishan National Nature Reserve (Fig. 2). Mean annual temperature between 1959 and 1988 ranged between −7 and −3 °C and mean annual precipitation between 700 and 1400 mm (Chen et al. 2011). The major geological groups at the sampled transect are alkalic pumice, trachyte, tuff and stomatal as well as laminated basalt.

The forests at the northern slope of Changbai Mountain have rarely been managed and therefore comprise mostly primary forest (Tang et al. 2011). Korean pine (*Pinus koraiensis* Siebold & Zucc.) intermixed with deciduous trees dominates between 800 and 1100 m. Above 1100 m up to 1700 m the dominant tree species is dark-bark spruce (*Picea jezoensis* var*. komarovii* Siebold & Zucc.), followed by Erman’s birch (*Betula ermanii* Cham.) at higher elevation. We established seven plots of an elevation difference of 150 m between plots, along this forest transect with every plot subdivided into eight subplots (Fig. 2). The sampling took place in early September 2019. From each subplot a 10 × 10 cm^2^ litter sample was taken, including the L- and F-layer. Microarthropods were extracted at room temperature using Berlese funnels (2 mm mesh size) for 5 days. After extraction, animals were stored in 75% ethanol.

### Species selection and body size measurement

**Fig. 3:**
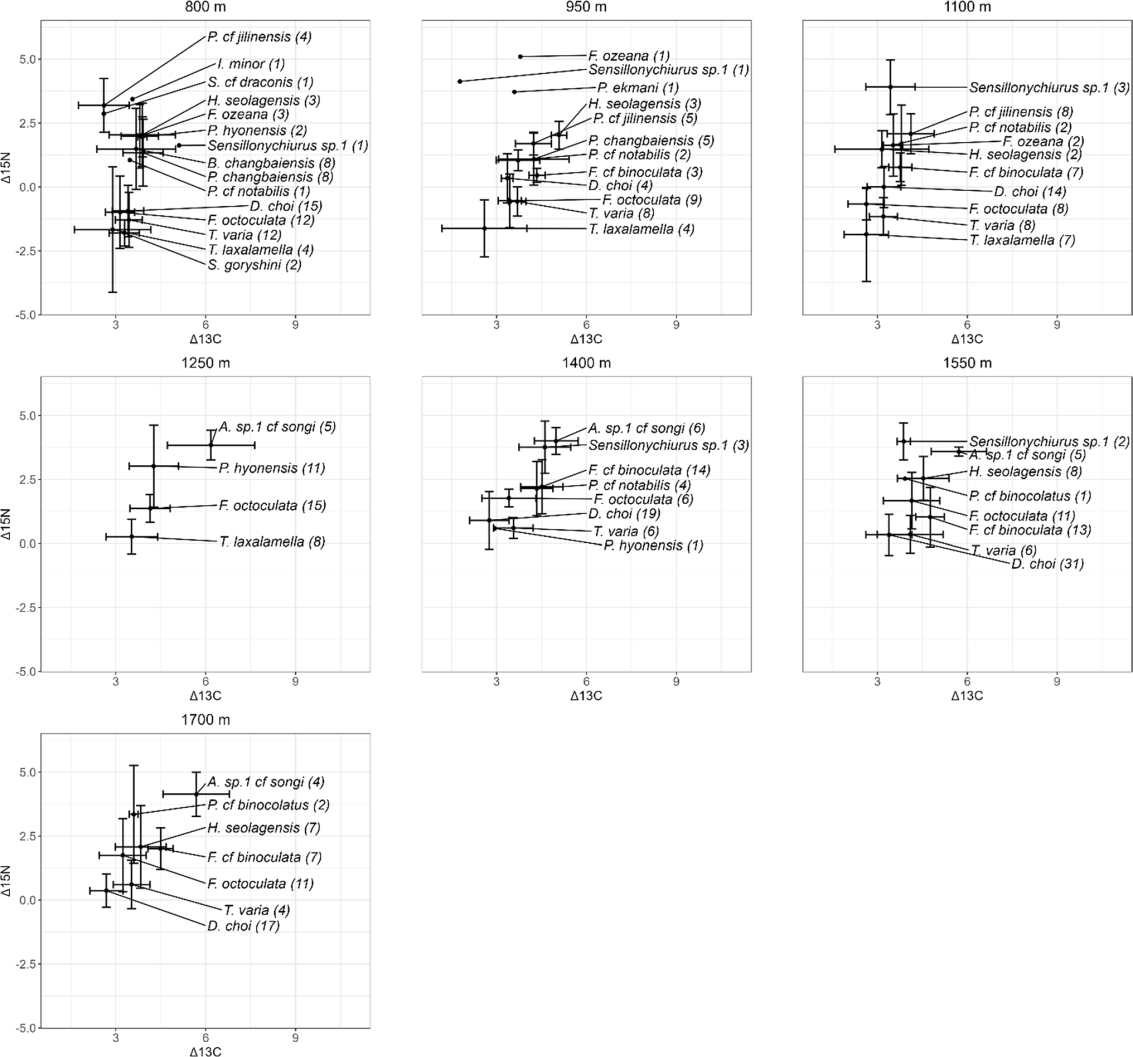
Stable isotope (Δ^15^N and Δ^13^C values) biplots of 19 Collembola species comprising 80% of the total abundance of Collembola at the respective elevation. Error bars represent standard deviations. Number of isotope measurements of species at the respective elevations are given in brackets

Collembola were determined under the microscope (Axio A1, Zeiss, Oberkochen, Germany). Specimens that needed further inspection, were either mounted in Hoyer’s solution or bleached in a 4:1 glycerine-lactic acid solution. Bleached individuals were not included in bulk stable isotope measurements. Individuals were determined at species or morpho-species level using relevant literature (Potapov 1991, 2001; Thibaud et al. 2004; Jordana 2012; Sun and Wu 2012; Yu et al. 2016; Potapov et al. 2018, 2020; Weiner et al. 2019; Sun et al. 2020; Sun 2021; Xie et al. 2019, 2022). Species accounting for the top 80% of total abundance per elevation were used for stable isotope analysis resulting in a total of 19 species. These species were assumed to be functional representatives of Collembola communities at our study sites. Prior to isotope measurements the body size of the specimens used was measured; if more than ten specimens were bulked only ten specimens representing the variation in body size within the respective bulk sample were measured. However, bulked specimens were of similar body length. This allowed calculating the mean body size of the Collembola measured, resulting in multiple measurements of the same species at the same plot as indicated in Fig. 3. Further, Collembola species were grouped into three life forms (Bitzer et al. 2005; Song et al. 2016; Xie et al. 2022), i.e. surface dwelling (epedaphic), litter dwelling (hemiedaphic) and soil dwelling (euedaphic) based on Gisin (1943), Hopkin (1997), Potapov (2001) and Widenfalk et al (2015). Regardless of their contribution to 80% total abundance, bulk stable isotopes of the three species that occurred at each of the seven elevations (*Desoria choi* Lee, *Folsomia octoculata* Handschin, *Tomocerina varia* Folsom) were measured to evaluate changes in stable isotope values of individual species with elevation.

### Stable isotope measurement

Stable isotope ratios of ^15^N/^14^N and ^13^C/^12^C were measured using an isotopic mass spectrometer (Delta V Advantage, Thermo Electron, Bremen, Germany) coupled via an interface (Conflo III, Thermo Electron, Bremen, Germany) to an elemental analyser (Euro EA 3000, EuroVector S. p. A. Milano, Italy). The relative abundances of ^15^N and ^13^C were expressed as $\updelta \left(\mathrm {\permil }\right)= \frac{{R}_{sample }- {R}_{standard}}{{R}_{standard}}\times 1000$, with R_sample_ and R_standard_ the ^15^N/^14^N or ^13^C/^12^C ratio in the sample and standard, respectively. Atmospheric N was used as standard for ^15^N, while Vienna Pee Dee belemnite for ^13^C. Acetanilide (C_8_H_9_NO) was used as internal standard. While stable isotopes of Collembola species were measured at every subplot, stable isotope values of litter were measured at every second subplot at every elevation; only leaf/needle litter without visible damage was used. To account for different types of litter we selected a representative fraction of the litter sample, dried and milled it, then an aliquot of this homogenized sample was measured. Stable isotope values were normalized to these δ values of litter from the respective elevation and subplot (Fig. 2) and expressed as Δ^15^N and Δ^13^C values. We used the mean δ values of litter of the two closest subplots to normalize Collembola from the subplots where δ values were not measured. This considered the high variability of the baseline among elevations and the (minor) variations between subplots within elevations (Fig. 2).

### Statistical analysis

All statistical analyses were performed in R v 4.0.4 (R Core Team 2021). Four one-dimensional metrics were calculated for each Δ^15^N and Δ^13^C values. Metrics included the abundance weighted isotopic positions (IPos Δ^15^N and IPos Δ^13^C), the isotopic range (Δ^15^N range and Δ^13^C range) as well as the maximum (Δ^15^N max and Δ^13^C max) and minimum (Δ^15^N min and Δ^13^C min) isotopic values at subplot level. Further, five multi-dimensional stable isotope metrics were calculated including both Δ^15^N and Δ^13^C values as described in Cucherousset and Villéger (2015). Prior to calculating multi-dimensional metrics, Δ^15^N and Δ^13^C values of Collembola species were abundance weighted as described above and then scaled between 0 and 1 to equalize contributions of the two isotopes. Five multidimensional metrics were calculated. (1) Isotopic divergence (IDiv) which approaches 1 if Collembola species with extreme isotope signatures are abundant and approaches 0 if they are rare. (2) Isotopic dispersion (IDis) which approaches 1 if abundant species have diverging isotope signatures, whereas it approaches 0 if abundant species have similar isotope signatures. (3) Isotopic evenness (IEve) which approaches 1 if species are evenly distributed in the isotopic space, whereas it approaches 0 if species cluster in a small area of the isotopic space. (4) Isotopic uniqueness (IUni) which approaches 1 if species occupy unique positions in the isotopic space, whereas it approaches 0 if species share similar isotopic niches. (5) Isotopic richness (IRic) representing the convex hull area spanning the total isotopic space of all species; IRic was not weighted by abundance since it represents functional diversity (Villéger et al. 2008). IRic approaches 1 if the hull area is large, whereas it approaches 0 if the hull area is small.

Linear relationships of the calculated multi- and one-dimensional metrics with elevation were analysed using linear models with the respective metric as response variable and elevation as ordered categorial variable. This way we tested for a linear trend using polynomial contrasts, if the test indicated a linear relationship elevation was transformed into a continuous independent variable. Elevation was left categorial if no such trend was found to test for general differences between elevations. Metrics were square-root-transformed if necessary (indicated in “Results”).

Further, “trait flex anovas” (Lepš et al. 2011) were calculated for Δ^15^N and Δ^13^C values. In short, this procedure allows to decompose the variation in community weighted Δ^15^N and Δ^13^C values across the elevation gradient explained by species turnover and intraspecific variation by calculating three linear models with varying response variables: The first contained the community weighted isotopes per subplot per elevation, henceforth termed specific averages, as response variable (similar to IPos Δ^15^N and IPos Δ^13^C, see above). Here, variation in the response variable may be caused by species turnover or intraspecific variability (or both). The second model contained the averages of isotopic averages of species across elevations weighted by their relative abundances per subplot per elevation, henceforth termed fixed averages. Here, variations in the response variable are only caused by species turnover. For the response variable of the third model, the fixed averages were subtracted from the specific averages. Here, variations in the response variable are only caused by intraspecific variability. Then, the sum of squares of the three models were decomposed as described in Lepš et al. (2011) to distinguish between the contribution due to species turnover, intraspecific variability and the covariation between the two.

Spearman Rank correlations of all metrics with six variables measured in the litter layer potentially linked to Collembola nutrition were tested. The fungal-to-bacterial PLFA ratio (fun/bac_litter_), the microbial biomass per gram organic carbon (C_mic_), the Gram^+^-to-Gram^−^ bacterial PLFA ratio (Gram^+^/Gram^−^), the saturated-to-monounsaturated PLFA ratio (sat/mono) and the cyclic-to-monoenoic precursor PLFA ratio (cyclo/pre) and the C/N ratio of the litter layer were included. Factors and their measurements are further characterized in Lux et al. (2022).

Linear mixed effect models were calculated using the “lme4” package (Bates et al. 2015) to analyse three common species present across all elevations. Unweighted Δ^15^N and Δ^13^C values of the respective species were used as response variable and elevation (continuous, selected as described above), body size and their interaction as independent variables. Body size and elevation were centred around their mean. To account for multiple measures at the same sampling location plotID was included as random intercept. Further, the influence of body size and Collembola life form on the isotopic values across the elevation gradient was investigated, including unweighted Δ^15^N and Δ^13^C values as response variable and elevation (continuous, selected as described above), mean body size, life from and their interaction as independent variables, again body size and elevation were centred around their mean. To account for multiple measurements of the same species at the same sampling location, plotID and species nested in plotID were included as random intercepts. To evaluate if mean body size of Collembola varies along the elevation gradient another linear mixed effect model was fitted, with body size as response variable and elevation (categorial, selected as described above) and life form as independent variables. The random intercept again included plotID and species nested in plotID. Body size (response variable) was log-transformed to increase homoscedasticity.

Significances of fixed effects were tested using type III sums of squares in the Anova function of the “car” package (Fox and Weisberg 2019). Adjusted R^2^ values are given in the respective regression figures. For linear mixed effect models Pseudo R^2^ of fixed effects (R^2^_fe_) and the whole model Pseudo R^2^ including the random effect (R^2^_total_) are given in the respective regression figures. Values and errors given in text represent the mean ± standard deviation. All linear models met the assumptions of homoscedasticity and Gaussian distribution of residuals.

## Results

### Stable isotope metrics along the elevation gradient

**Fig. 4:**
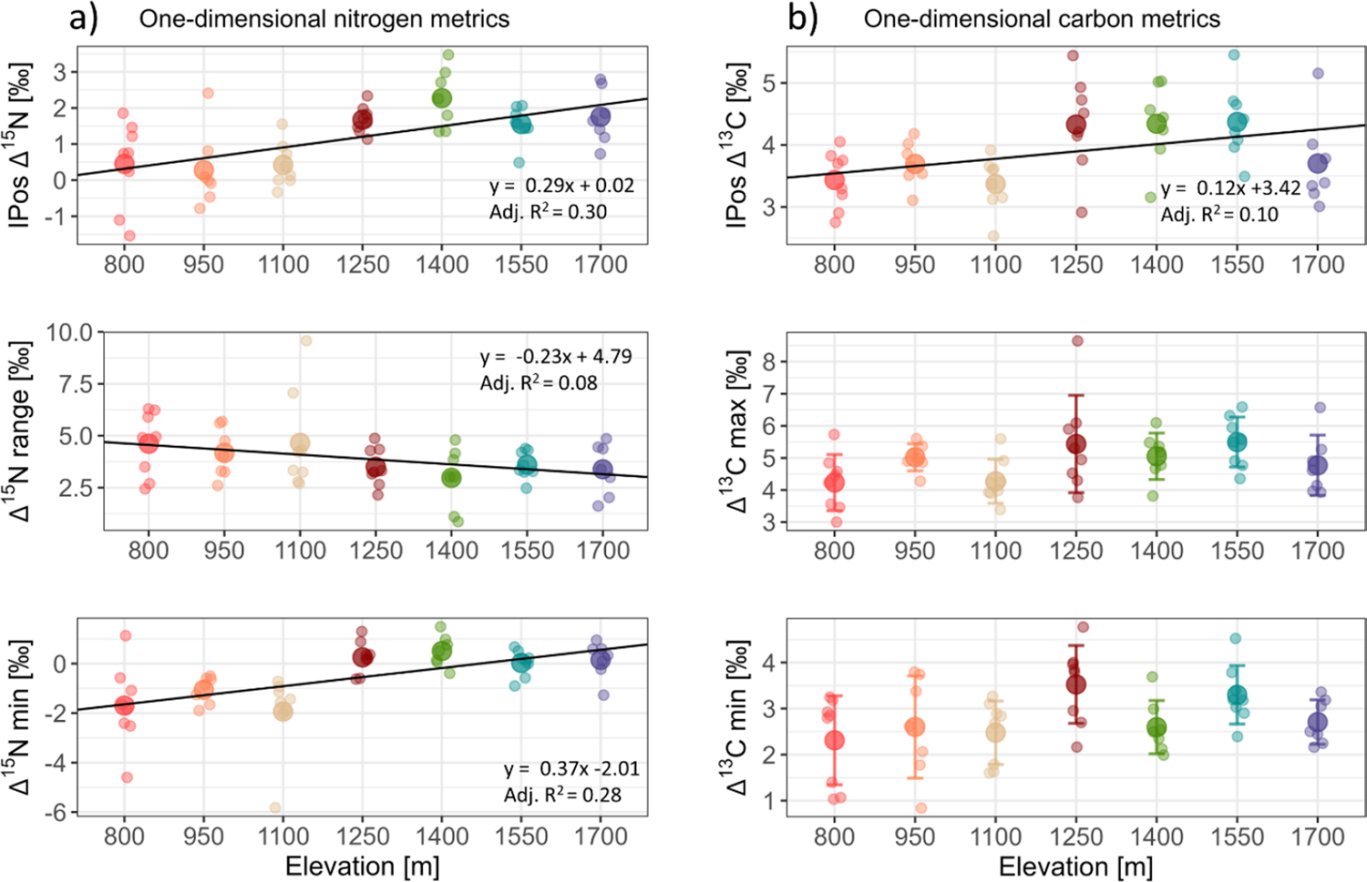
One- dimensional stable isotope metrics of nitrogen (**a**) and carbon (**b**), which significantly varied with elevation. Elevations are color coded; large dots represent the means and error bars standard deviations. Error bars are only displayed if the metric showed a non-linear response to elevation. Regression line formulas and adjusted R^2^ values (Adj. R^2^) are displayed for linear responses to elevation

Three one-dimensional metrics of Δ^15^N values, calculated from mean species stable isotope values (Fig. 3) at subplot level, differed significantly across the elevation gradient (Fig. 4a). The abundance weighted IPos Δ^15^N (F_1,51_ = 23.32, P < 0.001) increased from 800 (0.45 ± 1.21‰) to 1700 m (1.76 ± 0.76‰); 57.5% of the total variation explained by elevation was contributed by intraspecific variability and only 5.9% by species turnover, the other 36.7% were contributed by the covariation between the two. Further, Δ^15^N min increased with elevation (F_1,51_ = 20.98, P < 0.001), increasing from 800 (−1.70 ± 1.66‰) to 1700 m (0.15 ± 0.73‰, respectively). By contrast, Δ^15^N range decreased (F_1,51_ = 5.53, P = 0.023) from 800 (4.61 ± 1.56‰) to 1700 m (3.39 ± 1.25‰) (Fig. 4a). Further, three one-dimensional metrics of Δ^13^C values varied significantly with elevation (Fig. 4b).

The abundance weighted IPos Δ^13^C increased significantly (F_1,51_ = 6.70, P = 0.013) from 800 (3.43 ± 0.47‰) to 1700 m (3.70 ± 0.72; with values at 1250, 1400 and 1550 m higher than the value at 1700 m). As in Δ^15^N, intraspecific variability contributed most to the variation in Δ^13^C explained by elevation (35.6%), while species turnover only contributed 16.3% and the covariation between the two 48.1%. Additionally, Δ^13^C max and Δ^13^C min varied significantly with elevation but the response was not linear (F_6,46_ = 2.42, P = 0.041, F_6,46_ = 2.57, P = 0.031, respectively); both were lowest at 800 m (4.23 ± 0.87 and 2.30 ± 0.96‰, respectively), but Δ^13^C max was highest at 1550 m and Δ^13^C min at 1250 m (5.49 ± 0.78 and 3.52 ± 0.85‰, respectively).

**Fig. 5:**
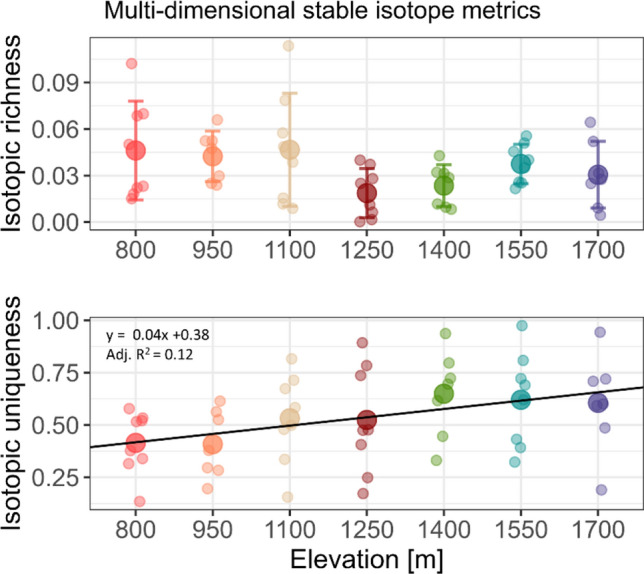
Multidimensional stable isotope metrics, which significantly varied with elevation. Elevations are color coded; large dots represent means and error bars standard deviations. Regression line formula and adjusted R^2^ value (Adj. R^2^) are displayed for the linear response to elevation. Isotopic richness was square-root transformed to approximate Gaussian distribution of residuals

Two multidimensional metrics of Δ^15^N and Δ^13^C values significantly varied with elevation (Fig. 5). IRic varied marginally significantly in a non-linear way with elevation (F_6,46_ = 2.08, P = 0.074, square-root-transformed data); it was lowest at 1250 m (0.02 ± 0.02; non-transformed mean) and highest at 1100 m (0.05 ± 0.04; non-transformed mean). IUni showed a linear trend, it increased with elevation from 800 m (0.41 ± 0.15) to 1700 m (0.61 ± 0.23) (F_1,51_ = 8.00, P = 0.007). A figure including non-significant isotopic metrics is given in the Appendix (Appendix Fig. 1).

IPos Δ^15^N and Δ^15^N min correlated significantly positive with the C/N ratio of the litter layer as well as C_mic_, sat/mono and cyclo/pre PLFA ratios (Table 1). Δ^15^N max on the other hand only correlated positively to cyclo/pre ratios across the elevation gradient. IPos Δ^13^C correlated significantly positive with C_mic_, sat/mono and cyclo/pre PLFA ratios. Δ^13^C min and Δ^13^C max both correlated positively with sat/mono and cyclo/pre ratios. Of the multidimensional metrics IRic correlated negatively with the litter C/N ratio and IUni correlated negatively with the fun/bac_litter_ across the elevation gradient.

**Table 1:** Spearman rank correlations between one- as well as multi-dimensional isotope metrics and litter characteristics [fungal-to-bacterial PLFA ratios in litter (fun/bac_litter_), microbial biomass (C_mic_), Gram + -to-Gram−, saturated-to-monounsaturated (sat/mono), and cyclic-to-monoenoic (cyclo/pre) PLFA ratios, C/N ratio] across elevations

	Factor	C/N	C_mic_	fun/bac_litter_	Gram^+^/Gram^−^	sat/mono	cyclo/pre
Nitrogen metrics	IPos Δ^15^N	**0.34**	**0.46**	0.02	0.04	**0.44**	**0.59**
	Δ^15^N range	−0.20	−0.15	−0.02	0.07	−0.19	−0.23
	Δ^15^N min	**0.47**	**0.51**	0.07	−0.06	**0.46**	**0.67**
	Δ^15^N max	0.20	−0.25	0.09	−0.05	0.16	**0.31**
Carbon metrics	IPos Δ^13^C	0.19	**0.30**	0.03	−0.09	**0.40**	**0.52**
	Δ^13^C range	−0.01	0.19	< 0.01	−0.02	< 0.01	0.09
	Δ^13^C min	0.10	0.14	0.15	−0.07	**0.36**	**0.39**
	Δ^13^C max	0.05	0.23	0.08	−0.04	**0.27**	**0.37**
Multidimensional metrics	Isotopic divergence	< 0.01	−0.06	0.08	−0.05	−0.14	−0.05
	Isotopic richness	−**0.32**	−0.04	−0.04	0.12	−0.22	−0.24
	Isotopic dispersion	−0.10	0.10	−0.11	0.27	< 0.01	−0.04
	Isotopic evenness	−0.10	0.09	−0.21	0.11	0.02	−0.09
	Isotopic uniqueness	0.02	0.23	−**0.34**	0.25	0.23	0.17

Values represent Spearman’s rho, values given in bold indicate Spearman’s rho to significantly differ from zero (P < 0.05)

### Response of Δ^15^N and Δ^13^C values of species to elevation

Underlining the high contribution of intraspecific variability to increased weighted IPos Δ^15^N with elevation, the unweighted Δ^15^N values of three species which occurred across the studied elevation gradient (*Desoria choi*, *Folsomia octoculata* and *Tomocerina varia*) increased significantly with elevation (χ^2^ = 10.19, P = 0.001; χ^2^ = 44.25, P < 0.001; χ^2^ = 22.70, P < 0.001, respectively). Δ^15^N values of *D. choi*, *F. octoculata* and *T. varia* increased from 800 (−0.94 ± 1.00‰, -0.78 ± 1.42‰ and −1.29 ± 1.07‰, respectively) to 1700 m (0.37 ± 0.65‰, 1.75 ± 1.43‰ and 0.61 ± 0.95‰, respectively; Appendix Fig. 2). Δ^15^N significantly decreased with body size only in *D. choi* (χ^2^ = 10.39, P = 0.001), it decreased by 0.48‰ per 1000 µm body size (Appendix Fig. 2). Unweighted Δ^13^C values of the three species, on the other hand, showed no linear trend with elevation nor with body size. Even though showing no linear response, Δ^13^C values generally varied with elevation in *D. choi and F. octoculata* (χ^2^ = 13.08, P = 0.042; χ^2^ = 28.81, P < 0.001, respectively). *D. choi* was lowest at 1700 m (2.69 ± 0.55‰) and highest at 800 m (3.41 ± 0.51‰). *F. octoculata* on the other hand was lowest at 1100 m (2.63 ± 0.60‰) and highest at 1250 m (4.15 ± 0.67‰).

### Response of Δ^15^N and Δ^13^C values of Collembola with life forms and body size

**Fig. 6:**
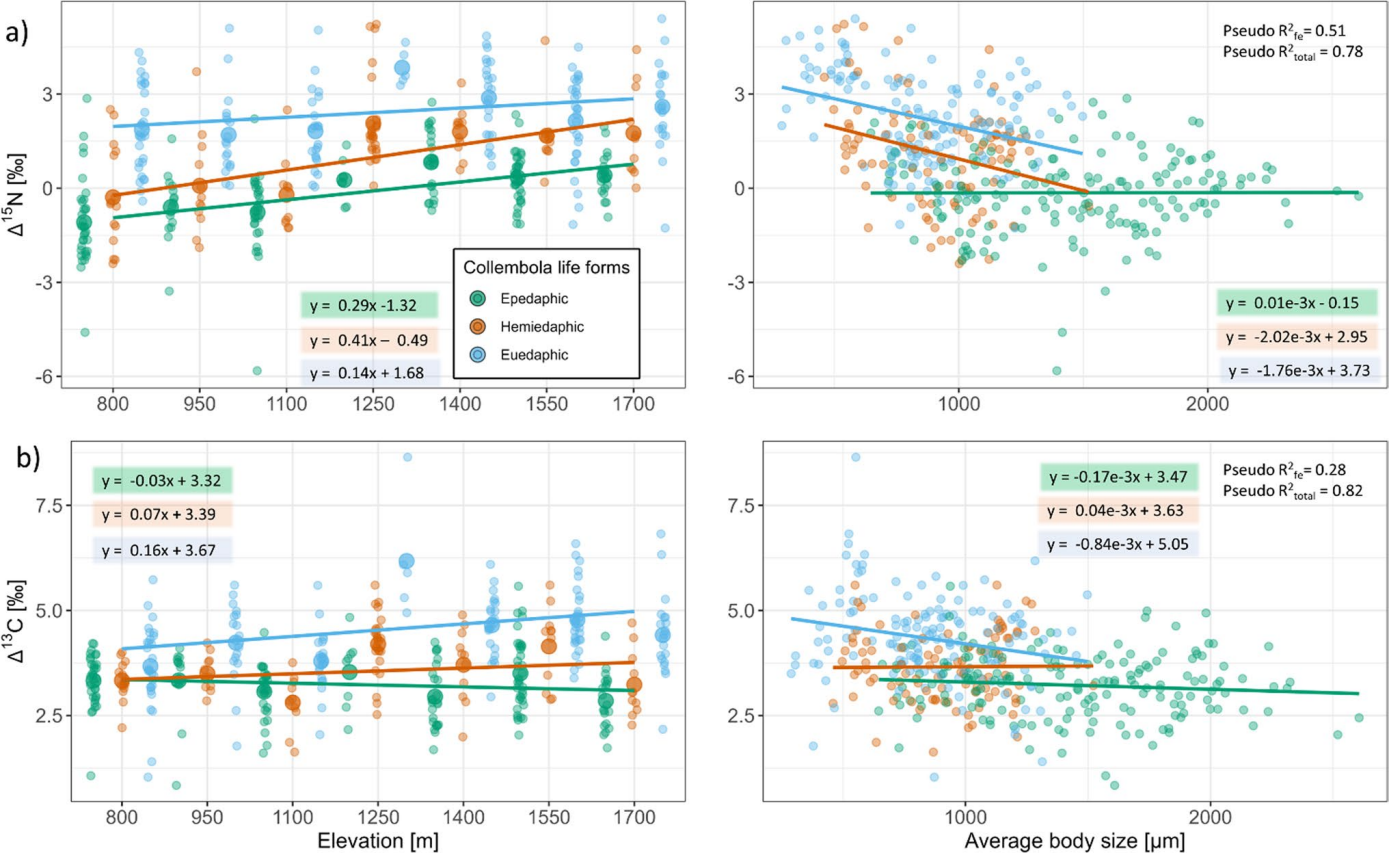
Δ^15^N (**a**) and Δ^13^C values (**b**) of different Collembola life forms (epedaphic, hemiedaphic and euedaphic) across elevations and body size. Colors mark life forms; means (large dots) and individual measurements (small dots). Pseudo R^2^ values refer to linear mixed effects models including Δ^15^N or Δ^13^C values as dependent variable, elevation (continuous), body size and life form as well as their interactions as fixed effects and plot ID as well as species nested in plot ID as random effect. For significant fixed effects and interactions see text

The unweighted Δ^15^N values of Collembola significantly increased with elevation (χ^2^ = 32.69, P < 0.001), varied with Collembola life form (χ^2^ = 130.15, P < 0.001) and decreased with mean body size of individuals per sample, henceforth body size (χ^2^ = 30.98, P < 0.001). Further, the response of Δ^15^N values to elevation depended on life form (significant elevation × life from interaction, χ^2^ = 28.98, P < 0.001; Fig. 6a). Δ^15^N values increased strongest between 800 and 1700 m in hemiedaphic Collembola (−0.52 ± 1.67‰ to 1.75 ± 1.42‰, respectively) and the increase was weakest in epedaphic Collembola (−1.09 ± 1.39‰ to 0.41 ± 0.70‰, respectively). The strength of the decrease of Δ^15^N with body size also depended on life form (significant life form × body size interaction, χ^2^ = 17.67, P < 0.001; Fig. 6a). Δ^15^N values decreased strongest with body size in hemiedaphic Collembola by 2.02‰ per 1000 µm body size, while there was no decrease in epedaphic Collembola with body size (< 0.01‰ per 1000 µm body size). Additionally, the interaction between body size and life form depended on elevation (significant elevation × life from × body size interaction, χ^2^ = 10.74, P = 0.005). Variations in the response of Δ^15^N values to body size with elevation were most pronounced in hemiedaphic Collembola where Δ^15^N values decreased by 2.26‰ per 1000 µm body size at 800 m, but only by 0.49‰ per 1000 µm body size at 1700 m.

Overall, the unweighted Δ^13^C values of Collembola significantly varied with Collembola life form (χ^2^ = 57.76, P < 0.001). Further, the linear response of unweighted Δ^13^C values to elevation depended on Collembola life form (significant elevation × life from interaction, χ^2^ = 8.01, P = 0.018). Δ^13^C increased strongest from 3.67 ± 1.02‰ to 4.41 ± 1.01‰ between 800 and 1700 m in euedaphic Collembola, while there was a slight decrease in epedaphic Collembola from 800 to 1700 m (3.33 ± 0.62‰ to 2.85 ± 0.64‰, respectively; Fig. 6b). Δ^13^C values decreased marginally significantly with increasing body size (χ^2^ = 3.45, P = 0.063) by 1.04‰ per 1000 µm body size. The interaction between life form and body size further depended on elevation (marginally significant elevation × life form × body size interaction, χ^2^ = 5.46, P = 0.065). Variations in the response of Δ^13^C values to body size with elevation were most pronounced in hemiedaphic Collembola where Δ^13^C values decreased by 0.65‰ per 1000 µm body size at 800 m, while showing an increase by 1.02‰ per 1000 µm body size at 1700 m.

Generally, body size varied with Collembola life form (χ^2^ = 207.97, P < 0.001, log-transformed data); epedaphic Collembola were larger (1452 ± 434 μm) than hemiedaphic (920 ± 237 μm) and euedaphic Collembola (857 ± 275 μm; untransformed means). Collembola body size varied across elevations followed a non-linear response (χ^2^ = 13.19, P = 0.040). However, differences in body size of life forms varied with elevation (significant elevation × life from interaction, χ^2^ = 32.81, P = 0.001; Appendix Fig. 3). The difference in body size between life forms was highest at 1250 m, where epedaphic Collembola were largest (1704 ± 210 μm), followed by hemiedaphic (883 ± 282 μm) and euedaphic Collembola (570 ± 35 μm).

## Discussion

Here, we investigated trophic shifts of Collembola communities across an elevation gradient using bulk stable isotope analysis at species level. The results showed that the trophic level of Collembola communities as well as selected Collembola species increased with elevation as indicated by IPos Δ^15^N values. Both life form (epedaphic, hemiedaphic and euedaphic) and body size influenced Δ^15^N and Δ^13^C values of Collembola, but their relative influence varied with elevation. Isotopic metrics including both Δ^15^N and Δ^13^C indicated that IRic was low at high elevation plots (1250–1700 m) and IUni increased with increasing elevation. Overall, our results indicate a decrease in Collembola acting as primary decomposers towards higher elevations where microbial resources prevail.

### Influence of elevation on trophic niches of Collembola

The trophic position of (functional) Collembola communities increased by about one third trophic level across the studied elevation gradient from 800 to 1700 m assuming an enrichment factor of 3.4‰ Δ^15^N per trophic level (Post 2002; Potapov et al. 2019b), which is in line with our first hypothesis. This shift is driven predominantly by intraspecific variations rather than species turnover. Large Collembola, such as *Tomocerus laxalamella* and *Tomocerina varia*, likely feed on litter and this is reflected by their negative Δ^15^N values at lower elevations. Negative Δ^15^N values of primary decomposers have been reported before for Collembola, Diplopoda and Oribatida (Scheu and Falca 2000; Pollierer et al. 2009; Schneider et al. 2004; Maraun et al. 2023) and presumably reflect that they feed on certain litter components depleted in ^15^N compared to bulk litter material, as shown for ^13^C of e.g., lignin or lipids (Pollierer et al. 2009). Even though lower Δ^15^N values may also indicate algal or lichen feeding (Maraun et al. 2023), NLFA analyses at the same sampling location and date indicate that algae are negligible as food resource for Collembola at Changbai Mountain (Lux et al. 2024). Lichen feeders usually have even lower Δ^15^N values as shown by Chahartaghi et al. (2005) who grouped Collembola species with Δ^15^N values similar to those of *T. laxalamella* and *T. varia* as primary decomposers. As Δ^15^N values of these putative primary decomposer species were most negative at lower elevations, the significant increase in minimum Δ^15^N values and the decreasing range in Δ^15^N values indicate a decrease in primary decomposers along the elevation gradient. Even though generally low across all elevations, IRic was especially low at higher elevations (1250–1700 m), which are characterized by pure coniferous forests. Decreases in primary decomposers consequently led to a smaller isotopic hull area.

IPos Δ^15^N values and Δ^15^N min positively correlated with C/N ratio of litter as well as microbial biomass, indicating that the shift from living predominantly as primary decomposer at lower elevation to living more as secondary decomposers at higher elevation, feeding either on living microorganisms or microbial residues, was driven by lower litter quality (i.e., higher C/N ratio). The negative correlation between IRic and C/N ratio also reflects this shift. Conform to this conclusion, Fujii et al. (2018) found the contribution of soil microarthropods to litter decomposition to be higher in nutrient rich than nutrient poor litter. By contrast, (Ma et al. 2019) concluded the contribution of soil fauna to litter (lignin) decomposition to be higher in more recalcitrant litter at Changbai Mountain. These contrasting results may implicate that soil fauna taxa other than Collembola are more involved in the fragmentation of structural litter compounds rich in lignin at higher elevations. However, the contradictory results could also be due to seasonal variations, which were not considered in our study and may be important for resource availability, especially at lower elevations where deciduous trees are more prevalent.

Generally, Collembola have been shown to preferentially feed on fungi (Pollierer and Scheu 2021; Li et al. 2022; Lux et al. 2024). However, as indicated by results of our study, a large fraction of the (functional) Collembola community may also predominantly feed on litter and only switch to feeding on resources of higher trophic level if nutrient limitation increases. This is also reflected by the correlation between Δ^15^N min (as well as IPos Δ^15^N) values of Collembola and the sat/mono and the cyclo/pre PLFA ratio in the litter layer, both reflecting nutritional and substrate-induced stress of microorganisms (Bossio and Scow 1997; Moore-Kucera and Dick 2008). Forests at higher elevations at Changbai Mountain are dominated by dark bark spruce (Liu 1997) and decomposition processes in spruce forests typically are slower than in deciduous forests (Albers et al. 2004; Berger and Berger 2012). Slow litter decomposition and associated microbial stress may have promoted the trophic shift in Collembola towards increased feeding on microbial resources at higher elevations. In plants it is well documented that increased stress results in increased herbivory (White 1993) and the same may hold true for microorganisms.

In addition to the increase in IPos Δ^15^N values with elevation, also Δ^15^N values of individual Collembola species, such as *Desoria choi*, *Folsomia octoculata* and *Tomocerina varia*, increased at higher elevation indicating that both Collembola communities, but also individual species, shifted their diet towards feeding more on microorganisms, microbial residues or other isotopic enriched resources at higher elevation. In fact, our study shows that most of the shift in community weighted trophic positions across elevations is due to intraspecific variations rather than species turnover. This is likely due to few very abundant species; for example, the trophic shift was particularly strong in *F. octoculata* indicating high trophic plasticity in this species. This is in line with findings of Hishi et al. (2007), who found this species to colonize different successional stages of litter. At Changbai Mountain, *F. octoculata* is among the most dominant Collembola species in forests across the studied elevation gradient (Xie et al. 2022). Its dominance across elevations likely also reflects its high trophic plasticity living as primary decomposer at lower elevations and as secondary decomposer at higher elevations, consuming microbes, microbial residues and/or other isotopic enriched resources. In fact, *F. octoculata* was also found to incorporate root-derived carbon (Fujii et al. 2016) and the higher trophic position at higher elevations therefore may be related to increased feeding on mycorrhizal fungi.

Besides these variations within species, the reported trophic shifts of Collembola communities were, at least to a certain extent, also due to species turnover. As shown recently, Collembola communities at Changbai Mountain are structured by the availability of food resources as indicated by litter and soil C/N ratios (Xie et al. 2022). Further, Collembola at high elevations likely occupy more unique trophic niches in the isotopic space, as indicated by the increase in IUni (Cucherousset and Villéger 2015). IUni was negatively correlated with the fun/bac_litter_ indicating that trophic niches additionally show a larger overlap if fungal resources become more abundant.

Therefore, our results suggest that trophic changes with elevation are driven by resource availability. The ability of Collembola to access resources in different microhabitats in the litter/soil matrix might consequently alter their trophic response to elevation (Erktan et al. 2020). The ability of accessing resources in the soil matrix is determined by Collembola life forms and their respective foraging strategies (Fig. 1). Among Collembola life forms, the shift in Δ^15^N values with elevation was strongest in hemiedaphic Collembola (including *F. octoculata*), with the values shifting from close to the ones in epedaphic species at 800 m towards the ones of euedaphic species at 1700 m, which is in line with our second hypothesis. Hemiedaphic Collembola likely are able to access similar resources as those used by euedaphic Collembola as body size in both groups was overall similar. Supporting the overlap in resource use by hemi- and euedaphic species, Fujii and Takeda (2012) found epedaphic Collembola species to predominantly colonize leaf litter placed on top of the soil in a coniferous forest, while leaf litter placed into the soils were colonized by both hemi- and euedaphic Collembola.

Even though Collembola life forms usually differ in their body size (Rusek 2007), body size variation within life forms likely also plays a role especially for species migrating vertically (Fig. 1). Accordingly, Δ^15^N values of hemiedaphic and euedaphic, but not in epedaphic Collembola decreased with body size. Presumably, this reflects that foraging strategies differ among Collembola life forms supporting our third hypothesis. As stressed repeatedly, traits of epedaphic Collembola, such as large body size, long furca and long antennae, hamper access to deeper soil layers, whereas smaller body size, and shorter (or absent) furca and shorter antennae of hemi- and euedaphic species allow access to food resources in smaller soil pores deeper in soil (Hopkin 1997; Erktan et al. 2020). Consequently, hemiedaphic Collembola may be able to shift resources towards those of euedaphic Collembola if necessary, i.e. if other food resources are lacking or are of poor food quality. Organic matter in deeper soil layers typically is enriched in ^15^N and ^13^C compared to litter material on top of the soil and this also likely is true for microorganisms (Wallander et al. 2004; Potapov et al. 2019a). Consequently, higher Δ^15^N values in hemi- and euedaphic than in epedaphic Collembola may, at least in part, be due to feeding on microorganisms or their residues deeper in soil (Potapov et al. 2019a). Supporting our conclusion that the switch of hemiedaphic Collembola to resources deeper in soil is due to poor litter resources at higher elevation, Xie et al. (2022) found the relative abundance of epedaphic species to decrease with increasing elevation at Changbai Mountain.

Feeding on litter resources may be facilitated by increased mandible size which is known to correlate with body size allowing to chew litter materials to get access to litter resources (Raymond-Léonard et al. 2019). Even though the relationship between body size and mandible function is more complex, the negative relationship between Δ^15^N values and Collembola body size support this inference, reflecting that litter material is more likely consumed by larger Collembola, while smaller Collembola preferentially consume microorganisms or microbial residues. However, epedaphic Collembola had low Δ^15^N values irrespective of body size, indicating that body size related variations in litter consumption are more important in hemi- and euedaphic species. This is also supported by the fact that Δ^13^C values of epedaphic Collembola were lowest among Collembola life forms and varied little with body size. However, as indicated by the interaction between body size, life form and elevation for both Δ^15^N and Δ^13^C, the response of trophic niches of Collembola to body size in different lifeforms varies between ecosystems, suggesting that it depends on local habitat conditions.

Interestingly, in some species, such as *F. octoculata* and *T. varia*, neither Δ^15^N nor Δ^13^C values significantly varied with body size, indicating that the trophic niche of these species does not change during development. However, in other species, such as *D. choi*, Δ^15^N values, but not Δ^13^C values, decreased significantly with body size indicating a shift from a more microbial-based diet in juveniles towards including more litter resources in adults. However, this shift in Δ^15^N was small despite the body size of *D. choi* varied considerably. Generally, the results indicate that trophic positions within Collembola species vary little with body size and thus developmental stages.

The increase in IPos Δ^13^C, Δ^13^C min and Δ^13^C max values along the studied elevation gradient was higher than expected from the increase in trophic position by one third trophic level (Post 2002) indicating that Collembola at higher elevations use resources more enriched in ^13^C. Similar to Δ^15^N values, Δ^13^C values of Collembola correlated positively with microbial biomass supporting the conclusion above that they more intensively consume microorganisms. In fact, Collembola at Changbai Mountain have been found to predominantly feed on fungi (Lux et al. 2024) and increased consumption of fungi deeper in soil by hemiedaphic Collembola may have contributed to the shift in Δ^15^N and Δ^13^C values of the Collembola community at higher elevation. Additionally, the increase in IPos Δ^13^C, Δ^13^C min and Δ^13^C max values of Collembola with increasing microbial stress underlines that adverse conditions in the litter layer at higher elevations contributed to the shift in resource use of Collembola.

## Conclusions

Results of the present study indicate that (functional) Collembola communities, as major decomposer animals in soil involved in litter decomposition, shift towards more feeding on microbes, microbial residues or even living as predators or scavengers at higher elevations. Trophically plastic species occurring at all elevations shifted towards higher trophic position and decomposing variations in Δ^15^N values indicated that the shift in Collembola communities towards higher trophic positions was mainly due to intraspecific shifts. The results further showed that the ability to access alternative food resources depends on Collembola life form and body size. The shift from lower to higher trophic positions with elevation suggests that global climate change, driving forest conversion, may be associated by a shift from decomposer soil microarthropods functioning as secondary decomposers towards functioning as primary decomposers in a warmer future climate, with potentially pronounced ramifications for decomposition processes, humus formation and carbon sequestration.

### Supplementary Information

Below is the link to the electronic supplementary material.

Supplementary file1 (PDF 706 kb)

## Data Availability

Δ^15^N and Δ^13^C, body size and life form of Collembola species as well as all calculated metrics are accessible via Dryad; https://doi.org/10.5061/dryad.8pk0p2nt1. During review please use: https://datadryad.org/stash/share/OOhQ-RdbvOFCDa573J2zMwtIjinpGnIuZ9EjDVFpFqo; Litter characteristics are accessible via Dryad as published by Lux et al. (2022); https://doi.org/10.5061/dryad.zs7h44jct.

## Code availability

We used established analyses, and metrics calculations were based on the published R script of Cucherousset and Villéger (2015), therefore no code is provided.
