# Supplementary material for: Trophic niches of Collembola communities change with elevation, but also with body size and life form
Source: Oecologia. 2024 Jan 24;204(3):491–504. doi: 10.1007/s00442-023-05506-7 (PMC10980659; doi:10.1007/s00442-023-05506-7)

Appendix

Journal: *Oecologia*

Publication title: Trophic niches of Collembola communities change with elevation, but also with body size and life form

Authors: Johannes Lux, Zhijing Xie, Xin Sun, Donghui Wu, Stefan Scheu

Appendix Figure 1: Non-significant one- and multidimensional metrics across elevation. Elevations are color coded; large dots represent the means and error bars standard deviations.

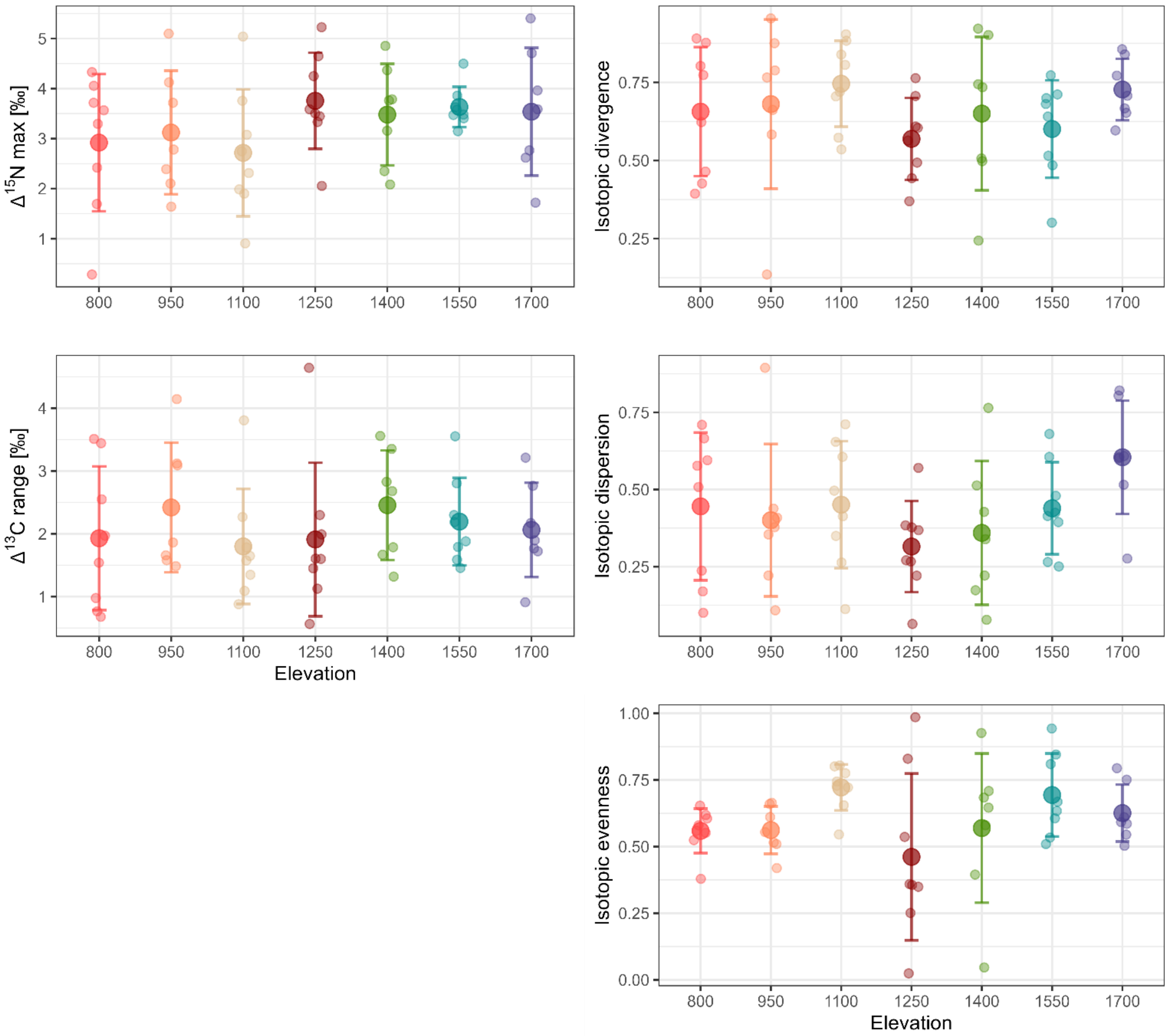

Appendix Figure 2: Δ^{15}N of *Folsomia octoculata*, *Desoria choi* and *Tomcerina varia* (marked in color) across the studied elevation gradient. Larger dots represent means, small dots individual measurements. Regression lines were plotted only for species in which a significant linear relationship was found. The pseudo R^2 values refer to linear mixed effects models including Δ^{15}N values of the respective species as dependent variable, elevation (continuous) and body size as fixed effect and subplot nested in elevation as random effect. For significant fixed effects see main text.

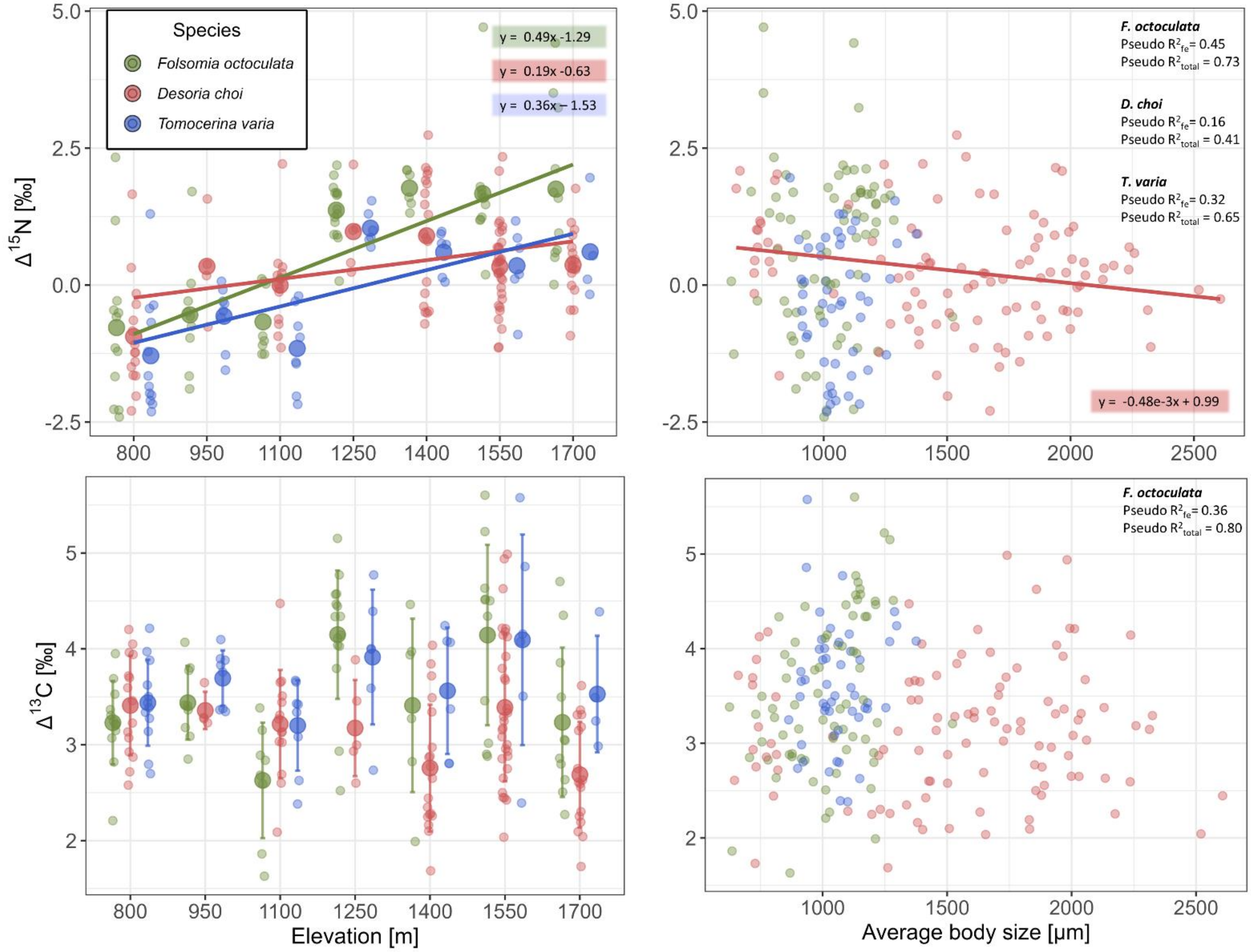

Appendix Figure 3: Body size [µm] of Collembola life forms (epedaphic, hemiedaphic and euedaphic; marked in color) across the studied elevation gradient. Large dots represent means, error bars standard deviations and small dots individual measurements. Dotted lines mark the mean body size across elevations for the respective life form. Note: The Y-axis displays the log-scale.

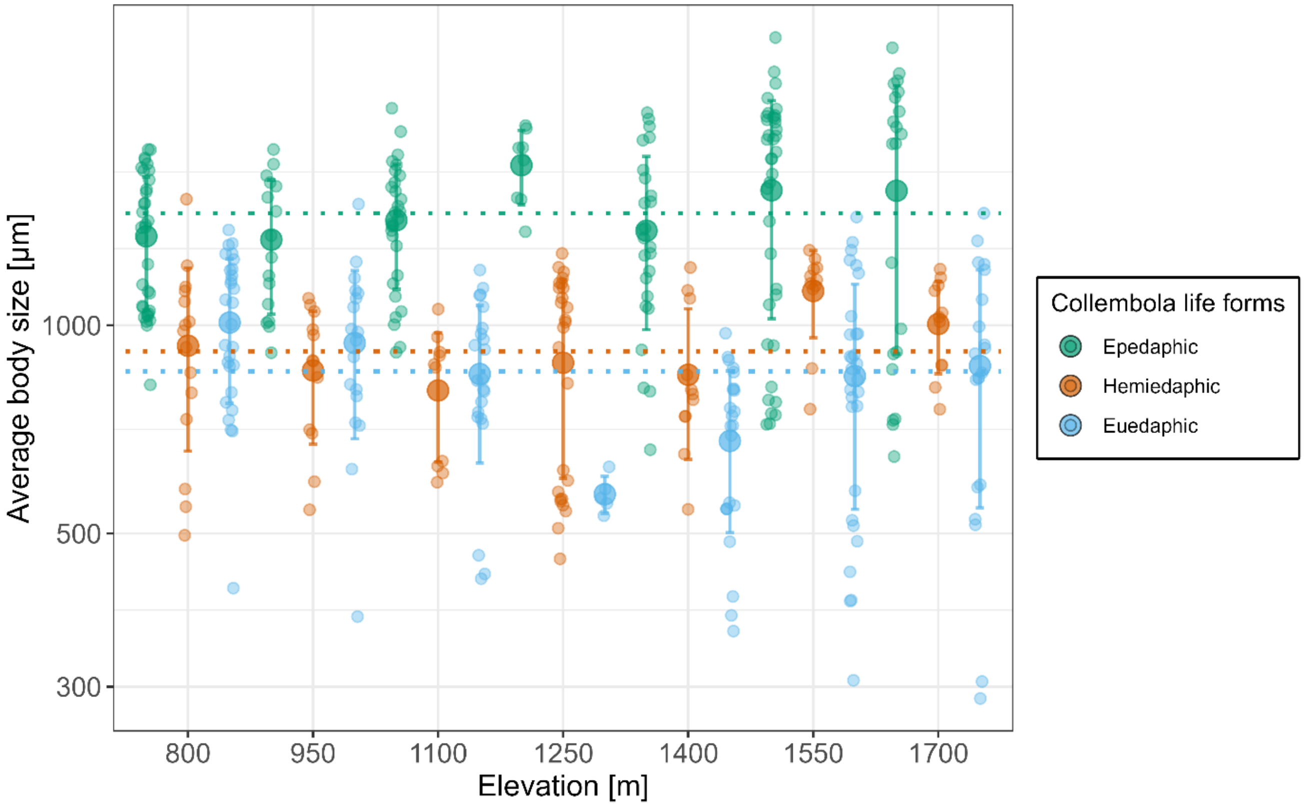

Supplement: Supplementary file 1 — Supplementary file1 (PDF 706 kb) [file 442_2023_5506_MOESM1_ESM.pdf]
